# Gender differences in nutrition literacy levels among university students and employees: a descriptive study

**DOI:** 10.1017/jns.2021.47

**Published:** 2021-07-30

**Authors:** Karianne Svendsen, Liv E. Torheim, Vibeke Fjelberg, Anita Sorprud, Ingunn Narverud, Kjetil Retterstøl, Martin P. Bogsrud, Kirsten B. Holven, Mari C. W. Myhrstad, Vibeke H. Telle-Hansen

**Affiliations:** 1 The Lipid Clinic, Department of Endocrinology, Morbid Obesity and Preventive Medicine, Oslo University Hospital, P.O. Box 4959 Nydalen, Oslo 0424, Norway; 2 Department of Nutrition, Institute of Basic Medical Sciences, Faculty of Medicine, University of Oslo, P.O. Box 1046 Blindern, Oslo 0317, Norway; 3 Department of Nutrition, Faculty of Health Sciences, Oslo Metropolitan University, P.O. Box 4 St Olavs Plass, Oslo 0130, Norway; 4 National Advisory Unit on Familial Hypercholesterolemia, Department of Endocrinology, Morbid Obesity and Preventive Medicine, Oslo University Hospital, P.O. Box 4959 Nydalen, Oslo 0424, Norway; 5 Unit for Cardiac and Cardiovascular Genetics, Oslo University Hospital, P.O. Box 4959 Nydalen, Oslo 0424, Norway

**Keywords:** Socioeconomic status, Education, Nutrition literacy, Public health, University, BMI, body mass index, CVD, cardiovascular disease, NL, nutrition literacy

## Abstract

The impact of nutrition information on public health is partly determined by the population's level of nutrition literacy (NL), which involves functional NL (such as knowledge of dietary guidelines) and critical NL (such as the ability to distinguish between evidence-based nutrition information and alternative facts). The aim of this cross-sectional study was to describe aspects of functional and critical NL and predictors of NL scores among university students and employees. We recruited at different university campuses, 414 students and 112 employees, of which 80 % were females and 69 % were in the ages of 18–30 years. In total, 82 % reported knowledge about where to find information on nutrition issues, and 70 % were familiar with Norwegian dietary guidelines. Being female, having higher age, being highly physically active and studying or working within health sciences were significant predictors of higher levels of functional nutrition knowledge. Significantly more women than men found it difficult to judge if media information on nutritional issues could be trusted (69 *v.* 54 %) and found it hard to distinguish between scientific and non-scientific information about diet (60 *v*. 42 %). Our findings indicate that for a sample of university students and employees, affiliation with health sciences, being female, having a higher age and being physically active were associated with higher functional NL. Women did, however, seem to have lower levels of some aspects of critical NL, e.g. how to critically judge nutrition information. A more thorough assessment of NL in university students and employees should therefore be conducted.

## Graphical abstract

**Figure:**
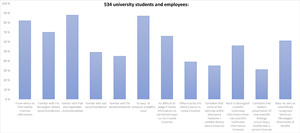

## Background

There is a well-known social gradient of cardiovascular disease (CVD) risk, where individuals with low socio-economic status (SES) display the highest risk factors for CVD compared to those with high SES^(1,2)^. Life expectancy, even within a city, can vary by 7–8 years between affluent and less affluent neighbourhoods^(3)^. Differences in health behaviour can explain some of the observed socio-economic gradients in CVD risk. Being a male college student with low SES has previously been associated with unhealthy dietary habits that increase the risk of premature CVD^(4)^. One possible mediator between low SES, health behaviour and CVD risk is health literacy. Health literacy has been defined by Berkman *et al.*^(5)^ as ‘the degree to which an individual can obtain, process, understand and communicate about health-related information needed to make informed health decisions’ (p. 16). Inadequate health literacy is strongly associated with low SES, poor health status, inactivity and overweight^(6)^. Health literacy has also been found to partially mediate the association between low education and health status^(7)^, or, with other words, high education is associated with high health literacy^(8)^. Despite this, the prevalence of inadequate health literacy was high in a highly educated Danish adult population^(4)^. In a recent International Health Literacy Population Survey (2019–2021), 33 % of 6000 Norwegians had a score at or below level 1, meaning that they lack essential key knowledge and skills necessary to take care of own health^(9)^. Adults with low health literacy levels are more likely to use non-scientific information sources such as television, social media, blogs or celebrity webpages than medical websites for health information^(10)^. The widespread use of such non-scientific medical health information among both young^(11)^ and older adults^(10)^ could therefore put public health at risk^(12)^. Nutrition literacy (NL) can be regarded as a sub-category of health literacy and involves functional skills (such as knowledge of dietary recommendations), interactive skills (the ability to communicate about nutrition and to translate nutrition knowledge into positive dietary choices), and critical NL including the ability to distinguish evidence-based nutrition information from alternative facts, among other aspects^(13,14)^. Interactive NL is less developed^(13)^, and thus the aim of the present study was to describe aspects of functional and critical NL among university students and employees and investigate possible associations with gender, age, body mass index (BMI), total cholesterol and field of study/work.

## Method

### Study design

In this cross-sectional study, we assessed demographics, two accessible CVD risk factors (BMI based on self-reported weight and height and point-of-care total cholesterol measurements) and aspects of functional and critical NL in students and employees at Oslo Metropolitan University (OsloMet), Norway. The data collection took place at two different campus locations for four consecutive days.

### Study sample

The study was advertised through the university web page and social media. Subjects who were pregnant, lactating or used cholesterol-lowering medication were not permitted to participate. In total, 548 university students and employees measured total cholesterol level, and 97 % (*N* 534) who also responded to the questionnaire were included in the study (Table 1). Participation was voluntary, and all participants gave verbal and written informed consent to the study.

**Table 1.** Background characteristics of the participants (*N* 534)

Variables	Total *N* 534		Women *n* 427		Males *n* 107		*P*^a^
Age group of 18–30 years, % (*n*/*N*)	69 (370/533)		69 (295/426)		70 (75/107)		0⋅865
Students, % (*n*/*N*)	79 (414/526)		80 (336/422)		75 (78/104)		0⋅303
Belongs to which faculty							
Faculty of Health Sciences, % (n/N)	48 (225/469)		50 (187/377)		41 (38/92)		0⋅164

Parent's country of origin		SD		SD		SD	
Western country ^b^,% (*n*/*N*)	85 (453/534)		86 (366/427)		78 (87/107)		0⋅020
Total cholesterol of >5 mmol/L, % (*n*/*N*)	17 (92/543)		14 (77/534)		14 (15/107)		0⋅325
Total cholesterol, mmol/L, mean,(sd	4⋅38	0.7	4⋅4	0.7	4⋅3	0.7	0⋅230
Have previously measured total cholesterol, yes (% (*n*/*N*)	24 (123/515)		24 (97/412)		25 (26/103)		0⋅725
BMI, kg/m^2^ , mean	23⋅7	3.5	23⋅5	3.5	24⋅6	3.2	0⋅002
Smokers^c^, % (*n*/*N*)	15 (80/519)		13 (55/413)		24 (25/106)		0⋅009
Using snuff, ^c^% (*n*/*N*)	19 (103/534)		17 (74/427)		27 (29/107)		0⋅022
Inactive^d^, % (*n*/*N*)	17 (88/529)		16 (68/426)		19 (20/103)		0⋅398
Family history of premature CVD, % (*n*/*N*)^e^	10 (50/528)		10 (41/422)		8 (9/106)		0⋅939
Medications^f^, % (*n*/*N*)	3 (15/517)		3 (11/420)		4 (4/105)		0⋅736

BMI, body mass index; *n*/*N*: *n* for the category sample and *N* for the total sample.

a *P*-value between genders using Pearson's *χ*^2^ for categorical variables and independent sample *t*-test for total cholesterol and BMI.

b At least one parent born in Western countries

c Smokers/using snuff = daily + occasional.

d Inactive=rarely or <1 time per week.

e Premature CVD = ≤55 years for men or ≤65 years for women among first degree relatives.

f Blood pressure lowering, diabetes medication and/or anticoagulating agents.

### Ethical approval

The study was approved by the Regional Ethical Committee of South-East with reference 2019/884 and the Norwegian Center for Research Data with reference 956205.

### Questionnaire

All participants completed a questionnaire with two parts: part 1 covered questions on age, gender, body weight and height, smoking habits, physical activity level, field of study/work (all self-reported) and measured total cholesterol level (registered in the questionnaire by study personnel) in addition to other background questions that are not presented herer. All questions except the field of study/work and years of education had previously been used in a similar setting in pharmacies among 18- to 90-year-old adults^(15)^.

Part 2 coveredtwelve questions regarding NL. The questions were selected from a validated Swiss short food literacy questionnaire by Krause *et al.*^(16)^ (functional NL, see Table 2) and a validated Norwegian NL questionnaire by Guttersrud *et al.*^(17)^ (critical NL, see Table 3). The Swiss questionnaire was translated to Norwegian by the research team, and some statements were changed to fit a Norwegian setting, whereas the questions in Norwegian remained unchanged. Six of the items that were taken from the Swiss questionnaire (NL1–NL6) focused on ‘functional NL skills’, such as the ability to obtain, process and understand basic nutrition information^(16)^, whereas six items from the Norwegian questionnaire (NL7– NL12**)** focused on ‘critical NL’^(17)^. Likert scales with four or five categories were used in both the original questionnaires, but the response categories in the present study were reduced to two options: 0 ‘disagree’ and 1 ‘agree’ to reduce the respondents’ load with filling in the questionnaire.

**Table 2.** Responses to functional NL statements by gender

	Percentage agreeing to the statement			
	Total (*N* 534)	Men (*n* 107)	Women (*n* 427)	*P*^a^
When I have questions on healthy nutrition, I know where I can find information on this issue (NL1)	82 %	84 %	81 %	0⋅49
I am familiar with the Norwegian dietary recommendations (NL2)	70 %	63 %	72 %	0⋅07
I know the official Norwegian recommendations about fruits and vegetable consumption (NL3)	88 %	73 %	92 %	<0⋅001
I know the official Norwegian recommendations about salt intake (NL4)	49 %	37 %	52 %	0⋅007
I know the official Norwegian recommendations about fat intake (NL5)	45 %	37 %	47 %	0⋅06
It is easy for me to compose a healthy meal at home (NL6)	87 %	86 %	87 %	0⋅83

NL; nutrition literacy.

a *P*-value between genders using Pearson's *χ*^2^. Questions NL1–NL6 were adapted to the Norwegian setting from Krause *et al.*^(16)^

**Table 3.** Responses to critical NL statements by gender

	Percentage agreeing to the statement			
	Total (*N* 534)	Men (*n* 107)	Women (*n* 427)	*P*^a^
It is difficult to judge if media information on nutritional issues can be trusted (inverse) (NL7)	66 %	54 %	69 %	0⋅004
I am influenced by the dietary advice that I read about in newspapers and magazines (inverse) (NL8)	39 %	27 %	42 %	0⋅005
I am confident that some of the methods within alternative medicine (such as health foods) provide me with credible dietary advice (inverse) (NL9)	35 %	25 %	38 %	0⋅011
I find it hard to distinguish scientific nutritional information from non-scientific nutritional information (inverse) (NL10)	56 %	42 %	60 %	0⋅001
I am confident that the media's presentation of new scientific findings concerning a healthy diet is correct (inverse) (NL11)	31 %	32 %	31 %	0⋅861
I base my diet on information that I get from scientifically recognised literature (for instance, the journals published by the Norwegian Medical Association and the Norwegian Directorate of Health) (NL12)	61 %	62 %	61 %	0⋅958

NL; nutrition literacy.

a *P*-value between genders using Pearson's *χ*^2^. Questions NL7–NL12 were obtained from Guttersrud *et al.*^(17)^ Inverse means that ‘agreeing’ indicates lower NLs.

### Total cholesterol measurement

We assessed total cholesterol levels in students and employees, as this is an important CVD risk factor that, at the same time, is easy and cheap to measure^(15,18)^. Total cholesterol was measured using finger-prick samples and the device Accutrend Plus (Roche Diagnostics) by trained study personnel. The measurement device captures total cholesterol levels in the range of 3⋅88–7⋅76 mmol/l. Values below 3⋅88 mmol/l were shown and reported as ‘low’ to the subjects and registered as 3⋅9 mmol/l, whereas values above 7⋅76 mmol/l were shown and reported as ‘high’ and registered as 7⋅8 mmol/l. After the measurements, all subjects received information on the impact of lifestyle on cholesterol levels verbally and, if they wanted more information, they received a brochure with healthy lifestyle advice. Subjects with total cholesterol of 7 mmol/l or higher were also advised to measure their total cholesterol level again during their next visit at their general practitioner.

### Statistics

Background data and functional and critical NL were presented stratified on gender and as means and standard deviations or % and *n*/*N*, and gender differences were tested using the *χ*^2^ test or the independent sample *t*-test.

The six functional NL questions (NL1–NL6, see Table 2) were found to have an adequate internal consistency, where the Kuder–Richardson 20 Test (KR20) (which is equivalent to Cronbach's *α* but for dichotomous variables) showed a KR20 reliability coefficient of 0⋅711. Thus, these questions were summarised into a functional NL scale. The critical NL questions, on the other hand, had a low KR20 reliability coefficient (0⋅445) and could therefore not be summarised to a score, and the items were examined separately in descriptive analysis.

For the functional NL scale, each affirmative answer to the statements (see Table 2 for statements) was given one point. The scale was thus from 0 to 6, where 6 was regarded as having the highest functional NL. The bivariate association between potential predictors and the functional NL scale was assessed using Spearman's correlation. Variables with a significance level of *P* ≤ 0⋅1 were included in an ordinal regression model to assess factors predicting functional NL. Data were analysed using SPSS Statistics 26 and STATA SE version 16.1.

## Results

### Background characteristics

Of the total sample of 534 students and employees, most (80 %) of the samples were female students in the age group of 18–30 years. Mean total cholesterol level and BMI were within the normal range in both men and women, but there were a higher proportion of men (27 %) than women (17 %) who used snuff and/or smoked (24 *v*. 13 %) (Table 1).

### Functional NL skills (NL1–NL6)

Of the total population, 82 % agreed to the statement that they knew where to find information on healthy nutrition issues (NL1). Furthermore, 70 % reported that they were familiar with the Norwegian dietary recommendations (NL2). Among specific food or nutrient recommendations, the majority (88 %) agreed that they were familiar with the recommendation about fruit and vegetable consumption (NL3), whereas the recommendation for salt intake (NL4) and fat intake (NL5) was less known (49 and 45 %, respectively). There was a higher proportion of women than men that reported to be familiar with all the specific nutrient recommendations (Table 2).

Bivariate analysis showed a positive correlation (*P* < 0⋅02) between the functional NL score and being female, above 30 years, being an employee (*v.* a student), studying or working at the Faculty of Health Sciences, being from a Western country, having a cholesterol level above 5 mmol/l, being a non-smoker and being physically active at least three to four times per week (data not shown). Having a BMI above 25 kg/m^2^ was however not associated with the functional NL score (and therefore not included in the ordinal regression model).

When controlling for the other variables in an ordinal regression model, being a student or employee at the Faculty of Health Sciences was associated with a higher likelihood of having a higher functional NL score (OR 1⋅50, 95 % CI 1⋅06, 2⋅11). Whereas males (OR 0⋅64, 95 % CI 0⋅42, 0⋅99), those 30 years of age and below (OR 0⋅39, 95 % CI 0⋅23, 0⋅65) and those being physically active less than three times per week (OR 0⋅05 95 % CI 0⋅35, 0⋅71) had significantly lower odds ratios for having a higher category of functional NL (Table 4).

**Table 4.** Factors associated with functional NL score (*N* 534)^a^

Variables	OR	95 % CI	*P*
Gender			
Female	1		
Male	0⋅64	0⋅42, 0⋅99	0⋅04
Age (years)			
>30	1		
18–30	0⋅39	0⋅23, 0⋅65	<0⋅001
Student or employee			
Employee	1		
Student	0⋅77	0⋅39, 1⋅51	0⋅45
Belongs to which faculty			
Not Faculty of Health Sciences	1		
Faculty of Health Sciences	1⋅50	1⋅06, 2⋅11	0⋅02
Parent's country of origin			
Non-Western country	1		
Western country	1⋅39	0⋅85, 2⋅26	0⋅19
Total cholesterol (mmol/l)			
>5	1		
≤5	0⋅83	0⋅51, 1⋅36	0⋅46
Smoking			
Yes	1		
No	1⋅31	0⋅83, 2⋅01	0⋅25
Physical activity			
Greater than or equal to three times per week	1		
Less than three times per week	0⋅50	0⋅35, 0⋅71	<0⋅001

a Adjusted odds ratios (OR) were derived from ordinal regression analysis in which each OR was adjusted for all other factors listed. An OR greater than 1 indicates that subjects with the characteristics have a higher likelihood of having a higher category of functional NL score.

### Critical NL skills (NL7–NL12)

As shown in Table 3, as many as 66 % of university students and employees agreed that they found it difficult to judge if information on nutritional issues from the media could be trusted (NL7), with a higher proportion among women (69 %) than men (54 %). Similarly, considerably more women (60 %) than men (42 %) found it hard to distinguish between scientific and non-scientific information about diet (NL10). With no gender difference, 31 % were confident that the ‘media's presentation of new scientific findings concerning a healthy diet is correct’ (NL11), whereas significantly more women (42 %) than men (27 %) agreed that they ‘let themselves be influenced by the dietary advice that they read about in newspapers’ (NL8). In total, 61 % stated that they ‘base their diet on information that they get from scientifically *recognized* literature including the national dietary recommendations’ (NL12). Nevertheless, a significant proportion of women (38 %) compared to men (25 %) agreed that alternative medicine could also provide credible dietary advice (NL9) (Table 3).

Straified into age groups, there was a larger proportion of ‘older' 31- to 70-year-old men (41 %) who stated that they were influenced by diet advice in the newspapers compared to those aged ≤30 years (younger) (21 %) (*P* 0⋅040). Concurrently, there was a lower proportion of older women (52 %) than younger women (63 %) who found it hard to distinguish between scientific and non-scientific nutrition information (*P* 0⋅022). No other differences in age groups were reported.

## Discussion

Most of the students and employees at a Norwegian university reported that they knew where to find information on nutrition issues and that they were familiar with the national dietary guidelines, indicating adequate functional NL skills. There seemed to be a lower proportion of men than women, agreeing that they were familiar with specific dietary recommendations, and being male mas associated with lower odds of having a high functional NL score. On the other hand, more women than men stated that (1) they were influenced by advice from the media and (2) they found it hard to distinguish between scientific and non-scientific nutrition information (critical NL).

### Functional NL

As many as 70 % of university students and employees reported to be familiar with the national dietary guidelines, mirroring what has been found in national surveys by the Norwegian Health Directorate^(19)^. The population overall seemed to be more familiar with the recommendations for fruits and vegetables than for the recommendations for intake of salt and fat, which is supported by Worsley *et al.*^(20)^. Nonetheless, the 30 % that disagreed, and were not familiar with the national dietary recommendations, could imply that even among university students and employees, there is a group that seemed to be resistant to the mass of nutrient and diet communication^(20)^. This warrants further studies to describe other characteristics with these students and employees. In the present study, we did not evaluate food literacy, which includes skills, such as to what degree can you apply the information for instance in regard to preparing food and ability to pick the healthy foods in the store^(13)^. Hence, although 70 % of the samples agree that they are familiar with the dietary recommendations, it cannot be translated to percent agreeing to follow the dietary guidelines. From a public health perspective, it is also challenging that many consumers choose less healthy eating options without being aware that it is in fact the less healthy option^(21)^.

We found that being female, having a higher age, studying or working at the Faculty of Health Sciences, and being more physically active were significant predictors of higher levels of functional NL in our sample of university students and employees. Supporting our findings, an Australian study found that higher age, female gender and university education were among the major predictors of higher nutrition knowledge score^(20)^. However, high education does not automatically imply high nutrition or health literacy; a population study among Danes found that despite being a relatively highly educated group of individuals, the prevalence of inadequate health literacy was high^(6)^. In our study, being overweight, having a higher total cholesterol level and being a smoker did not seem to impact the functional nutrition knowledge score. In comparison, overweight but not smoking was associated with low health literacy in the Danish adults^(6)^. These diverging findings could partly be due to our population being healthier than in the Danish study: in both students and employees, BMI and total cholesterol level were on average within the recommended levels. The prevalence of smokers and those who used snuff were also in line with the general Norwegian population as reported by Statistics Norway^(22)^.

### Critical NL

We also assessed aspects of critical NL, which implies that one is able to judge nutrition information, and to critically reflect on the factors that influence dietary behaviour for the individual and the society^(13)^. We found that 82 % agreed that they knew where to find information about healthy nutrition. In comparison, a considerable proportion (37 %) found it challenging to find health-promoting information, which is in line with the results in a recent report on health literacy level in a representative sample of the Norwegian population^(9)^. Furthermore, about 30 % of both men and women reported that they were ‘confident that the media's presentation of new scientific findings concerning a healthy diet is correct’, which could be worrying considering the other finding that 56 % of the same population of highly educated women and men had problems in distinguishing between scientific and non-scientific nutrition information. Supporting this result, a large proportion of the previously mentioned comparable Norwegian population found it challenging to assess whether they could trust information from the mass media about diseases (55 %) and health risks (49 %)^(9)^.

We observed several gender differences in the assessment of critical NL. First, there was a significantly higher frequency of women (60 %) than men (42 %) who found it hard to distinguish between scientific and non-scientific information about diet. Next, more women than men responded that they were influenced by dietary advice presented in the media (42 *v.* 27 %, respectively) and found alternative medicine advice to be credible (38 *v*. 25 %, respectively). Since only 20 % of the study samples were men, these observed gender differences warrant further studies. The newly published report on the Norwegian population's health literacy level^(9)^ showed that only 20 % reached a high level of general health literacy (covering aspects such as find, understand, assess and use health-related information). Furthermore, that women and those with an education above upper secondary level had slightly better skills^(9)^An assessment of the population's NL level still remains to be elucidated in Norway.

### Strengths and limitations

The major strength of our study was that we were able to include a considerable proportion of students and employees during one week of recruitment. This was probably due to the low threshold for participation on different university campus locations and the inclusion of total cholesterol measurements. Although we attempted to include students and employees from several fields of study, the majority of the final sample belonged to the Faculty of Health Sciences. Furthermore, 80 % were females, and mean total cholesterol levels and BMI were within the normal range, indicating that we included a health-conscious sample.

There are some limitations with the NL questions (part 2 of the questionnaire). First, the questionnaire concerning NL has not been validated but was rather combined by selected questions from two validated questionnaires^(16,17)^. Secondly, the critical NL questions could be directly used since the questionnaire was in Norwegian^(17)^, but the functional NL questions selected from a questionnaire by Krause *et al.*^(16)^ had to be translated. The latter questions^(16)^ were not back-translated after having been translated to Norwegian. Furthermore, in the present study there were only two response categories (agree or disagree), whereas the original questionnaires had five response categories. Hence, there might have been nuances that we did not capture. These are limitations that should be taken into account when interpreting the results. Furthermore, for future studies, a tool covering all three NL domains should be developed and validated in Norway, preferable using Rasch modelling^(17)^.

## Conclusion

We found that university students and employees appeared to have a relatively high functional NL knowledge (e.g. of dietary guidelines and where to find nutrition information) and that predictors of a higher functional NL score were being female, having higher age, being physically active and affiliated with health sciences. Whereas the main challenge, particularly among women, seemed to be in the critical NL domain such as difficulties in distinguishing between scientific and non-scientific information about diet, despite that 50% of the sample was affiliated with health sciences. The present study therefore calls for a thorough assessment of functional and critical NL levels among university students and employees. If our present results are verified, actions to increase critical NL levels to avoid misinformation among university students and employees should be taken.

## References

[1] McNamara CL, Balaj M, Thomson KH, (2017) The socioeconomic distribution of non-communicable diseases in Europe: findings from the European Social Survey (2014) special module on the social determinants of health. Eur J Public Health 27, 22–26. 10.1093/eurpub/ckw222 28355638

[2] Stringhini S, Carmeli C, Jokela M, (2017) Socioeconomic status and the 25×25 risk factors as determinants of premature mortality: a multicohort study and meta-analysis of 1⋅7 million men and women. The Lancet 389, 1229–1237. 10.1016/S0140-6736(16)32380-7 PMC5368415 28159391

[3] Vale PH (2020) Life expectency in Oslo regions vary-why? Det norske medicinske Selskab 2, 453–476.

[4] Martinez-Lacoba R, Pardo-Garcia I, Amo-Saus E, (2018) Socioeconomic, demographic and lifestyle-related factors associated with unhealthy diet: a cross-sectional study of university students. BMC Public Health 18, 1241. 30404595 10.1186/s12889-018-6149-3 PMC6223081

[5] Berkman ND, Davis TC & McCormack L (2010) Health literacy: what is it? J Health Commun 15, 9–19. 10.1080/10810730.2010.499985 20845189

[6] Svendsen MT, Bak CK, Sørensen K, (2020) Associations of health literacy with socioeconomic position, health risk behavior, and health status: a large national population-based survey among Danish adults. BMC Public Health 20, 565. 32345275 10.1186/s12889-020-08498-8 PMC7187482

[7] van der Heide I, Wang J, Droomers M, (2013) The relationship between health, education, and health literacy: results from the Dutch Adult Literacy and Life Skills Survey. J Health Commun 18, 172–184. 24093354 10.1080/10810730.2013.825668 PMC3814618

[8] Michou M, Panagiotakos DB, Lionis C, (2019) Socioeconomic inequalities in relation to health and nutrition literacy in Greece. Int J Food Sci Nutr 70, 1007–1013. 30935258 10.1080/09637486.2019.1593951

[9] Le C, Finbråten HS, Pettersen KS, (2020) Befolkningens helsekompetanse. del I. Oslo Helsedirektoratet.

[10] Chen X, Hay JL, Waters EA, (2018) Health literacy and use and trust in health information. J Health Commun 23, 724–734. 30160641 10.1080/10810730.2018.1511658 PMC6295319

[11] Klassen KM, Douglass CH, Brennan L, (2018) Social media use for nutrition outcomes in young adults: a mixed-methods systematic review. Int J Behav Nutr Phys Act 15, 70. 30041699 10.1186/s12966-018-0696-y PMC6057054

[12] Naeem SB, Bhatti R & Khan A (2020) An exploration of how fake news is taking over social media and putting public health at risk. Health Info Libr J 12, 1–7. 10.1111/hir.12320 PMC7404621 32657000

[13] Krause C, Sommerhalder K, Beer-Borst S, (2018) Just a subtle difference? Findings from a systematic review on definitions of nutrition literacy and food literacy. Health Promot Int 33, 378–389. 27803197 10.1093/heapro/daw084 PMC6005107

[14] Velardo S (2015) The nuances of health literacy, nutrition literacy, and food literacy. J Nutr Educ Behav 47, 385-9.e1. 26026651 10.1016/j.jneb.2015.04.328

[15] Svendsen K, Jacobs DR, Jr., Royseth IT, (2019) Community pharmacies offer a potential high-yield and convenient arena for total cholesterol and CVD risk screening. Eur J Public Health 29, 17–23. 30239673 10.1093/eurpub/cky190

[16] Krause C G, Beer-Borst S, Sommerhalder K, (2018) A short food literacy questionnaire (SFLQ) for adults: findings from a Swiss validation study. Appetite 120, 275–280. 28912107 10.1016/j.appet.2017.08.039

[17] Guttersrud O, Dalane J & Pettersen S (2014) Improving measurement in nutrition literacy research using Rasch modelling: examining construct validity of stage-specific ‘critical nutrition literacy’ scales. Public Health Nutr 17, 877–883. 23472785 10.1017/S1368980013000530 PMC10282317

[18] Catapano AL, Graham I, De Backer G, (2016) ESC/EAS Guidelines for the Management of Dyslipidaemias: the task force for the Management of Dyslipidaemias of the European Society of Cardiology (ESC) and European Atherosclerosis Society (EAS) developed with the special contribution of the European Assocciation for Cardiovascular Prevention & Rehabilitation (EACPR). Atherosclerosis 2016, 281–344. 10.1016/j.atherosclerosis.2016.08.018 27594540

[19] Helsedirektoratet (2018) Utviklingen i norsk kosthold 2018. Oslo: Helsedirektoratet.

[20] Worsley A, Wang WC, Byrne S, (2014) Different patterns of Australian adults’ knowledge of foods and nutrients related to metabolic disease risk. J Nutr Sci 3, e14. 25191606 10.1017/jns.2014.12 PMC4153087

[21] van Buul VJ, Bolman CAW, Brouns F, (2019) Use of nutritional information: analysing clusters of consumers who intend to eat healthily. J Nutr Sci 8, e17. 31080590 10.1017/jns.2019.13 PMC6498756

[22] Statistics Norway (2017). *Tobacco, Alcohol and Other Drugs*. Available at: https://www.ssb.no/statbank/table/05307/tableViewLayout1/ (accessed 13 March 2019)

